# Speed management across road environments of varying complexities and self-regulation behaviors in drivers with cataract

**DOI:** 10.1038/s41598-022-10952-z

**Published:** 2022-04-28

**Authors:** Sonia Ortiz-Peregrina, Carolina Ortiz, Francesco Martino, Miriam Casares-López, José J. Castro-Torres, Rosario G. Anera

**Affiliations:** Department of Optics, Laboratory of Vision Sciences and Applications, University of Granada, Edificio Mecenas, Av. Fuentenueva s/n, 18071 Granada, Spain

**Keywords:** Diseases, Engineering, Optics and photonics

## Abstract

Evidence suggests that drivers with cataract self-regulate their driving, but there is a lack of objective information. This study compared speed behavior in older drivers with and without cataract and how the parameter is influenced by road traffic complexity and driver characteristics. The study included 15 drivers with cataract and a control group of 20 drivers. Visual status was assessed using visual acuity, contrast sensitivity, and intraocular straylight. Speed management was studied using a driving simulator. Driving difficulty and self-regulation patterns were evaluated by means of the Driver Habits Questionnaire (DHQ). The cataract group showed a significant decrease in visual function in all the parameters evaluated (*p* < 0.05). These drivers tended to drive at lower speeds than the control group. Road characteristics, gender, and intraocular straylight in the better eye were identified as significant predictors of speed management. Drivers with cataract experience greater driving difficulty, particularly when driving at night (*p* < 0.05). Drivers with cataract reduce their driving speed more than older drivers without visual impairment. The straylight parameter may be a good indicator of each driver’s subjective perception of their own visual ability to drive. This work helps shed light on the mechanisms through which age-related visual impairment influences driving behavior.

## Introduction

Driving is considered essential for maintaining independence and quality of life, so its discontinuation brings negative health consequences^1,2^. Self-regulation is the strategy by which aging drivers try to compensate for a loss of key functional abilities, such as declining sensory, cognitive, and motor capacities, which can compromise their ability to drive safely^3^. Visual impairment is one the most concerning causes of self-regulation or modified driving in older drivers^4^. A leading cause of age-related vision decline is the loss of transparency in the lens of the eye, which commonly progresses towards a cataract. Cataract account for 33% of visual impairments worldwide^5^. It is predicted that 25% of drivers will be 65 or older by the year 2030^6^, dramatically increasing the number of drivers with cataract on the road^7^. This condition is typically bilateral and seriously compromises visual acuity and contrast sensitivity, while also increasing disability glare^8^. Patients with cataract tend to spend considerable periods driving in these conditions before undergoing cataract surgery. Meanwhile, their driving performance is negatively affected^9,10^, and the risk of an accident is estimated to increase by a factor of 2.5^11^. Drivers with cataract may be aware of their limitations, so they often restrict or self-regulate their driving, avoiding challenging situations such as driving at night, in heavy traffic, or during adverse weather conditions^12^. Previous studies have generally only identified self-regulating drivers via self-reported data collected from questionnaires or travel diaries. However, a growing body of evidence shows that such questionnaires may not be a reliable means of quantifying self-regulation practices^13–15^. A recent study therefore compared self-regulation data obtained from the Driving Habits Questionnaire (DHQ) and naturalistic driving data with the authors concluding that the only situation presenting any agreement was the habit of avoiding night-time driving^15^.

Although older drivers adopt certain modifications in their driving behavior and habits, the vast majority continue to drive. While driving, they may also demonstrate awareness of the risk associated with their limitations and modify their style by reducing their speed, for example. This is supported by self-reported data which shows that older drivers usually avoid high-speed roads^2^. It has been suggested that speed selection is based on interactions between the driver, the vehicle, and the road traffic environment^16^. This hypothesis has not been studied in older drivers, either with or without a visual impairment such as cataract, so there is a lack of empirical evidence regarding the mechanisms underlying speed behavior in these drivers. Reducing speed in response to a visual decline is logical, because it gives drivers more time to integrate visual information and respond to environmental features (e.g., a sharp curve), traffic signals, or unexpected events (e.g., a jaywalking pedestrian).

Visual fitness to drive is predominantly assessed using the visual acuity test. However, research in drivers with a real or simulated cataract has questioned the test’s validity as a predictor of driving performance, accident risk, or probability of driver self-regulation^2,4,10,11,17^. On the other hand, there is also evidence that other non-standardized visual tests such as the useful field of view (UFOV), contrast sensitivity, straylight, and glare-based tests could be more accurate in detecting which of these drivers could be at risk^4,9–11,17–19^.

The aim of this study was to compare speed behavior among older drivers with and without cataract and determine the influence of road traffic complexity and driver characteristics (i.e., age, gender, and visual status) on speed self-regulation*.* Of the driver attributes, we intended to determine which visual function parameters, if any, could accurately predict speed behavior. Lastly, self-reported driving difficulty was used to compare both groups of drivers as a subjective measure of how cataract may influence driver self-regulation.

## Methods

### Participants

The sample consisted of 15 older drivers with cataract and 20 older drivers with no visual impairment (control group). The age of the cataract group ranged from 56 to 77 years (65.53 ± 6.31 years) and 27.7% were female. Ten participants had bilateral cataract and five unilateral cataract. All were recruited from the San Cecilio University Hospital (Granada, Spain) and diagnosed by the same ophthalmologist. For grading the disease, we used the classification system proposed by Artal et al., (2011) based on OSI (objective scatter index) measurement. This parameter is measured objectively using the OQAS II (Optical Quality Analysis System II, Visiometrics SL, Tarrasa, Spain) optical device, based on the double-pass technique^20,21^. According to this system, eyes are classified as normal (OSI < 1), early cataract (1.0 ≤ OSI < 3.0), mature cataract (3.0 ≤ OSI < 7.0) and severe cataract (7.0 ≤ OSI). From the 30 eyes of the cataract group, 5 (16.7%) had no cataract (monocular cases), 10 (33.3%) had early cataract, 8 had mature cataract (26.7%) and 7 had severe cataract (23.3%).

Participants in the cataract group had no other eye diseases, no previous eye surgeries, and a binocular visual acuity ≥ 20/40 (the minimal VA required to hold a driving license in Spain) with their regular optical correction worn for driving. The control group were aged from 56 to 68 years (61.75 ± 3.80 years) and 5% were female. They were free of any eye diseases. Both groups were age matched, and all participants were in good general health and licensed drivers who drove regularly. Participants completed a questionnaire about their driving characteristics before enrolling in the study.

The study was conducted with approval from the University of Granada Human Research Committee (921/CEIH/2019) and a signed informed consent form was obtained in accordance with the Declaration of Helsinki. Visual and driving performance was measured with participants wearing the optical correction usually worn for driving (if any), with the addition of an appropriate working distance lens if required.

### Visual assessment

Distance visual acuity (VA) was evaluated with the POLA Vista Vision (DMD Med Tech srl., Torino, Italy) acuity chart at 5.5 m (logMAR scale). Contrast sensitivity (CS) was evaluated using the CSV-1000 test (Vector Vision, Ohio, USA), a reliable tool for measuring this visual function parameter^22^. The test operates at spatial frequencies of 3, 6, 12, and 18 cycles per degree (cpd) and was performed at the recommended distance of 2.5 m. Results were expressed as log units.

Intraocular straylight is defined as the veil of luminance over the retina generated by light scattered by the eye media. Straylight affects visual function, causing disability glare and hazy vision^23^. We measured this parameter using the C-Quant (Oculus DG, Germany) computer-controlled straylight meter, which employs a compensation comparison method^24–26^. The measurements were taken in a darkened room and values were expressed as logarithm of straylight (log(s)), such that higher values indicate a greater influence of straylight.

Visual outcomes were expressed for the better and the worse eye separately, defined in terms of visual acuity^8^.

### Driver self-regulation

#### Driving simulator: speed management

**Figure 1:**
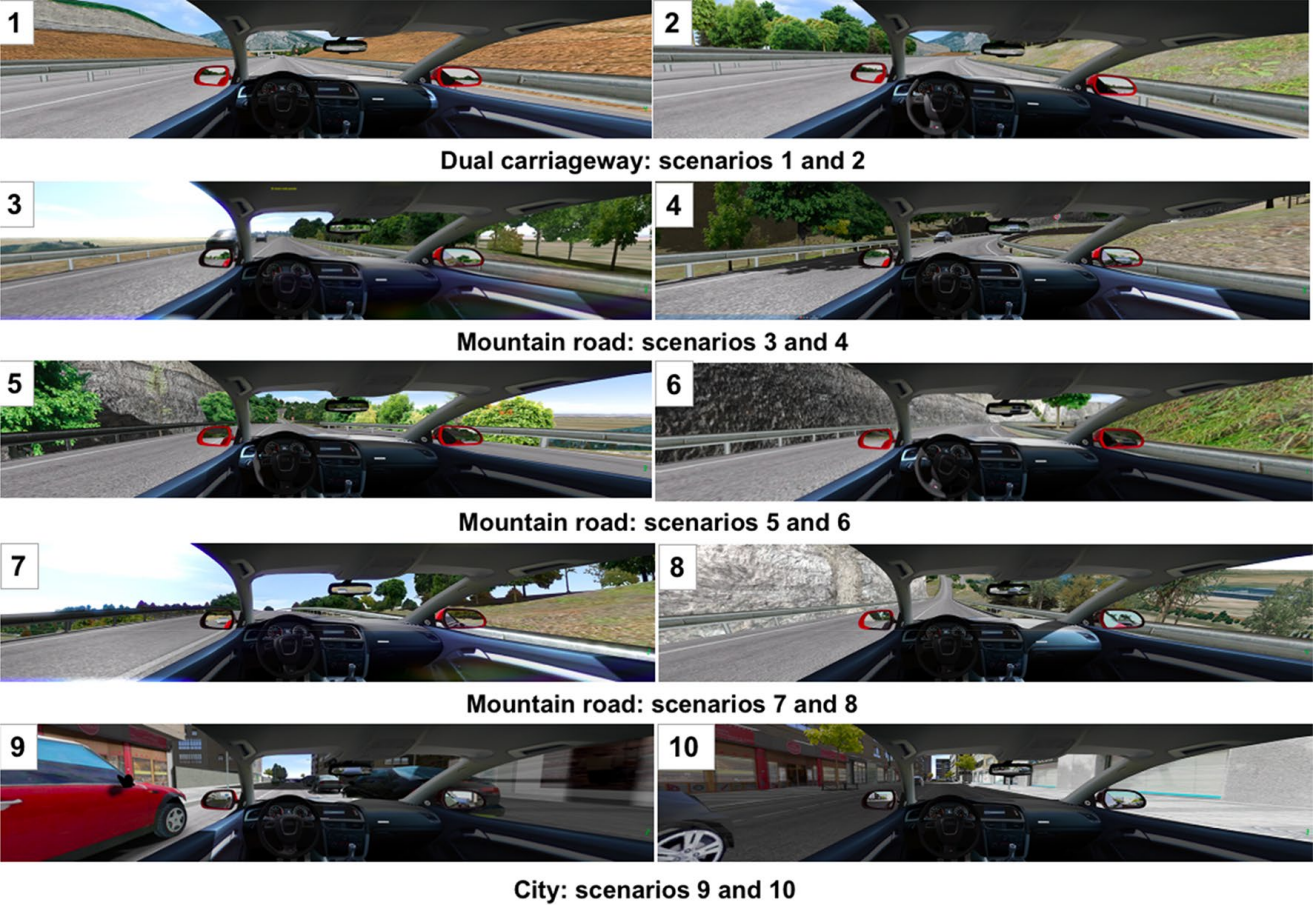
Screenshots of the different scenarios used in the analysis.

As an objective method of assessing driver self-regulation practices, speed behavior in different conditions was measured using a driving simulator as in previous studies^9,27–29^. Participants drove a route of approximately 12.5 km and took approximately 15 min if they drove close to the speed limits. The simulation included three different road types with speed limits and a design similar to those found on the Spanish road network: dual carriageway, mountain road, and inner-city. Within these road types, different scenarios were chosen to analyze the influence of different road characteristics on driving speed. The scenarios had varying degrees of traffic complexity (oncoming cars, cars in the same direction), different layouts (straight, slight bends, sharp bends), and the presence/type of slope (no slope, ascending, descending). The inner-city route also included the presence of parked cars at the sides of the road. Table 1 and Fig. 1 show the characteristics for each scenario.

**Table 1:** Characteristics of the different driving scenarios used in the analysis.

Scenario	Road type	Speed limit (kph)	Road geometry and traffic complexity			
			Other traffic	Road geometry		Parked cars
				Road layout	Slope	
1	Dual carriageway	120	Same direction	Straight	No	No
2				Slight bend		
3	Mountain road	90	Oncoming/Same direction	Straight	Gentle/ascending	No
4				Sharp bend		
5	Mountain road	40	Oncoming/Same direction	Straight	Gentle/ascending	No
6				Sharp bend		
7	Mountain road	90	Oncoming/Same direction	Straight	Steep/ascending	No
8					Steep/descending	
9	City	50	Same direction	Straight	No	Yes
10						No

To analyze the speed data, we selected a representative 100 m section for each driving scenario, with different characteristics in terms of road geometry, speed limit, and traffic complexity. This length meant that both traffic conditions and road geometry were as uniform as possible throughout the section being analyzed, thus guaranteeing that driving performance was studied under specific conditions. Furthermore, there was a sufficient separation between the various sections with different characteristics used in the analysis. This ensured that each section did not bear an influence on the next one because drivers were still adapting their driving to the previous scenario. This procedure for section analysis has been employed in previous studies^28,30,31^.

Before the experiment, participants received at least two training sessions in similar environments to those used in the experimental drive, with a 1-week interval between sessions. They were instructed to drive as they normally do, without imposing a minimum speed or obedience to speed limits to gain insight into their natural driving behavior. Along the route, there were a total of 41 speed limit signs that looked just like those on real roads. The digital speedometer, located on the central display, was visible at all times. If any symptoms of simulator sickness were noted, the session was stopped, and the subject was excluded from the study.

#### Driving habits questionnaire (DHQ)

The Driving Habits Questionnaire (DHQ) was designed to collect information about driving in the last 12 months^11^. Eight items score the degree of visual difficulty experienced in specific driving situations. Scores were based on a 5-point scale (5 = no difficulty, 4 = a little difficulty, 3 = moderate difficulty, 2 = extreme difficulty, 1 = so difficult that I no longer drive in that situation). A composite score of driving difficulty on a 100-point scale can be obtained from the responses to the items (composite score = (mean score − 1) × 25). Lower scores represent a greater level of difficulty.

### Statistical analysis

Statistical analyses were performed using SPSS 24.0 (SPSS Inc., Chicago, IL) and statistical significance was set to a p-value < 0.05. Descriptive statistics were used to summarize all variables of interest (demographics, driving habits, visual function parameters, and driving speed). Group comparisons were made for visual function parameters and speed management across driving scenarios using the independent t-test or Mann–Whitney U test when normality could not be assumed. We used a generalized linear mixed model (GLMM) with repeated measures to identify which road/driver characteristics influenced the participants’ speed behavior. The model included mean speed as the dependent variable and driving scenario as the repeated factor. Driver characteristics (age, gender, and visual function parameters) were also included as possible predictors of speed. This model has previously been validated as an approximation of driving performance^28,30^. Mean scores for every item in the DHQ and the composite score were compared between groups using the Mann–Whitney U test.

## Results

Table 2 summarizes participants’ driving characteristics for both groups. All of them rated their driving ability as normal to good and all were current drivers, with most of them driving daily or several times a week. In the control group, most participants drove more than 5000 km in the previous year, and between 1000 and 4999 km in the cataract group. With regard to self-perceived driving ability, it is striking that more participants in the cataract group rated their driving as good than in the control group.

**Table 2:** Descriptive results of driving characteristics.

Group driving characteristics	Control (%)	Cataract (%)
Number of accidents in the past year	0	0
**Self-perceived driving ability**		
Excellent	11.1	0
Good	61.1	83.3
Normal	27.8	16.7
Fair or bad	0	0
**Driving frequency**		
Daily	44.4	50
Several times a week	50	28.6
Once a week	5.6	14.3
2–3 times a month	0	0
Once a month	0	7.1
**Distance driven in the last year (km)**		
500–999	0	7.1
1000–4999	23.5	71.4
> 5000	76.5	21.4

### Group comparison of visual function outcomes and speed management across driving scenarios

Drivers with cataract exhibited significantly impaired visual function compared to the control group for all the parameters studied. Differences were significant for both the worse eye and the better eye (Table 3).

**Table 3:** Comparison of visual outcomes for drivers with and without cataract (Mann–Whitney U test).

	Control	Cataract	*p*-value
**Better eye**			
VA (logMAR)	− 0.02 ± 0.06	0.21 ± 0.20	< 0.001
Log CS	1.69 ± 0.13	1.16 ± 0.49	< 0.001
Log(s)	1.21 ± 0.16	1.50 ± 0.23	< 0.001
**Worse eye**			
VA (logMAR)	0.01 ± 0.04	0.56 ± 0.36	< 0.001
CS	1.62 ± 0.15	0.65 ± 0.47	< 0.001
Log(s)	1.17 ± 0.11	1.73 ± 0.26	< 0.001

The worse and better eye were defined according to visual acuity.

On the other hand, the results obtained for speed management show that subjects with cataract generally drove more slowly (Table 4). The only scenario for which participants with cataract drove slightly faster was scenario 3, but this difference was about 1.0 kph. The t-test results indicated that the cataract group drove significantly slower for some scenarios: scenario 1 (dual carriageway, slight bend, 120 kph speed limit [SL]), scenario 4 (mountain road, sharp bend, 90 kph SL), scenario 6 (mountain road, sharp bend, 40 kph SL), scenario 7 (mountain road, ascending slope, 90 kph SL), and scenario 8 (mountain road, descending slope, 90 kph SL). In scenario 5 (mountain road, straight, 40 kph SL), both groups drove above the legal speed limit. In scenario 8, only drivers in the control group drove above the limit.

**Table 4:** Comparison of speed self-regulation in drivers with and without cataract for scenarios of varying driving difficulty.

Scenario	Control, speed (mean ± DS) (kph)	Cataract, speed (mean ± DS) (kph)	Control, speed–speed limit (mean ± DS) (kph)	Cataract, speed–speed limit (mean ± DS) (kph)	Mean difference	t	*p*-value
1	119.33 ± 12.98	102.55 ± 21.48	− 0.67 ± 12.98	− 17.45 ± 21.48	16.78	2.681	0.014
2	110.66 ± 12.50	98.92 ± 19.17	− 9.34 ± 12.50	− 21.08 ± 19.17	11.74	2.066	0.05
3	76.52 ± 16.47	77.62 ± 10.14	− 13.48 ± 16.47	− 12.38 ± 10.14	− 1.1	− 0.226	0.822
4	62.88 ± 8.51	50.14 ± 6.89	− 27.12 ± 8.51	− 39.86 ± 6.89	12.74	4.748	< 0.001
5	46.61 ± 14.88	44.53 ± 7.43	6.61 ± 14.88	4.53 ± 7.43	2.08	0.495	0.624
6	39.71 ± 5.00	35.22 ± 6.38	− 0.29 ± 5.00	− 4.78 ± 6.38	4.49	2.333	0.026
7	70.93 ± 8.51	58.80 ± 9.26	− 19.07 ± 8.51	− 31.20 ± 9.26	12.13	4.021	< 0.001
8	91.46 ± 13.47	76.58 ± 18.33	− 1.46 ± 13.47	− 13.42 ± 18.33	14.88	2.773	0.009
9	29.27 ± 12.20	24.45 ± 13.88	− 20.73 ± 12.20	− 25.55 ± 13.88	4.82	1.089	0.284
10	29.62 ± 13.75	24.06 ± 11.69	− 20.38 ± 13.75	− 25.94 ± 11.69	5.56	1.261	0.216

Mean speed and mean speed adaptation with respect to the speed limit are included.

### Influence of road complexity and driver characteristics on speed behavior

The GLMM model revealed some significant predictors of speed management for older drivers (Table 5). With respect to road scenarios and traffic complexity, scenario 10 was chosen as the reference category as it was considered the simplest segment along the route. Thus, the model indicated that almost all the scenarios had characteristics that were significant predictors of speed. For all the dual carriageway and mountain road scenarios (scenarios 1 to 8), estimates showed higher speeds than in the reference category. Estimates of driving speed were lower for scenarios with a curved layout than those with a straight layout and the same speed limit. On the other hand, the straight segments with slopes (scenarios 7 and 8) were also associated with higher speeds than the reference category. The slope that caused the highest speeds was the descending section. Finally, in the urban environment, the presence of vehicles parked did not seem to influence the speed adopted by the participants.

**Table 5:** Generalized linear mixed model (GLMM) estimates of speed self-regulation.

Parameter	Coefficient	SE	t-statistic	*p*-value	95% CI
**Road scenario/complexity**					
Scenario 1: dual carriageway, straight, 120 kph SL	84.68	3.72	22.777	< 0.001**	[77.23, 92.14]
Scenario 2: Dual carriageway, slight bend, 120 kph SL	78.20	3.45	22.683	< 0.001**	[71.30, 85.10]
Scenario 3: Mountain, straight, 90 kph SL	49.61	3.35	14.795	< 0.001**	[42.90, 56.31]
Scenario 4: Mountain, sharp bend, 90 kph SL	30.15	2.45	12.329	< 0.001**	[25.24, 35.06]
Scenario 5: Mountain, straight, 40 kph SL	18.51	3.00	6.163	< 0.001**	[12.51, 24.51]
Scenario 6: Mountain, sharp bend, 40 kph SL	10.31	2.33	4.415	< 0.001**	[5.62, 15.00]
Scenario 7: Mountain, straight, ascending, 90 kph SL	38.42	2.60	14.753	< 0.001**	[33.21, 43.63]
Scenario 8: Mountain, straight, descending, 90 kph SL	57.16	3.37	16.955	< 0.001**	[50.41, 63.90]
Scenario 9: City, straight, parked cars, 50 kph SL	-0.11	2.98	-0.037	0.971	[-6.07, 5.85]
Scenario 10: City, straight, no parked cars, 50 kph SL	–	–	–	–	–
**Driver characteristics**					
**Age**	− 0.04	0.14	-0.261	0.795	[-0.32, 0.24]
**Gender**					
Male	5.61	2.32	2.414	0.017*	[1.03, 10.20]
Female	–	–	–	–	–
**Visual parameters**					
**Better eye**					
VA	7.82	6.21	1.259	0.209	[− 4.43, 20.07]
CS	4.72	3.03	1.554	0.122	[− 1.27, 10.70]
Log(s)	− 13.68	6.05	− 2.261	0.025*	[− 25.61, − 1.75]
**Worse eye**					
VA	− 7.91	5.74	− 1.378	0.170	[− 19.24, 3.41]
CS	− 1.20	4.01	− 0.299	0.765	[− 9.11, 6.71]
Log(s)	4.85	5.45	0.887	0.376	[-5.93, 15.63]
Intercept	32.35	11.14	2.904	0.004*	[10.39, 54.31]
Number of observations	350				
Akaike information criterion	2607.01				
Bayesian information criterion	2644.76				

Reference category (-); *p < 0.05; **p < 0.001.

**Figure 2:**
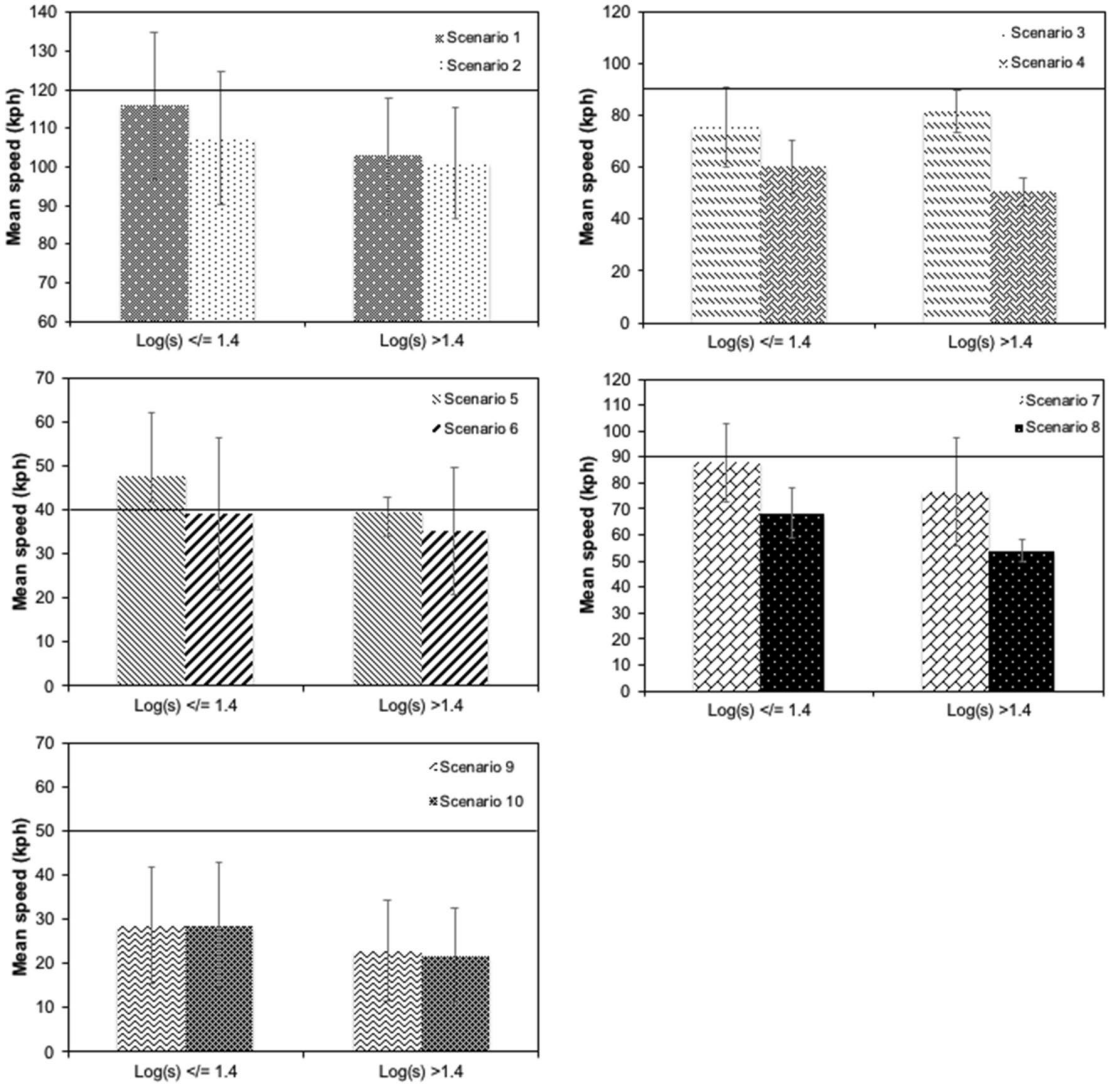
Mean speed of drivers in different scenarios with respect to the log(s) level in the better eye. Log(s) groups are based on the cut-off limit (log(s) = 1.4). Speed limit for scenarios on each case is marked with a solid line.

Some driver characteristics were also found to be significant predictors of driving speed. Although age did not have an influence, we found that gender influenced speed selection. Men drove faster than women, with a difference of 5.61 kph. Intraocular straylight in the better eye also influenced driver speed. According to the model, an increase of one unit in the log(s) of the better eye (a worse value) implied a speed reduction of 13.68 kph. Figure 2 shows the mean speeds measured in the different scenarios according to the level of straylight in the better eye. As such, it is evident that participants with a log(s) of greater than 1.4 drove more slowly in all scenarios except for scenario 3 (mountain road, straight, 90 kph SL). Other authors have proposed this value of log(s) as the cut-off value for safe driving^24,32,33^.

### Self-perceived driving difficulty: driver habits questionnaire (DHQ)

Table 6 presents the results of self-perceived difficulty for each driving situation with respect to group (control and cataract). There are certain tasks for which drivers with cataract did not perceive any more difficulty than the control subjects, such as left turns in traffic or driving on freeways. The cataract group experienced more perceived difficulty for the rest of the items. Between them, driving at night stands out, as this was the only item that showed significantly lower scores in the cataract group. Apart from self-regulating their driving by reducing their speed, some participants in the cataract group indicated that they do not drive alone, on rainy days, in heavy traffic or during darkness. This last situation was the most avoided scenario for drivers with cataract (25%). Finally, the composite score was significantly higher for the control group (*p* = 0.025), which suggests that the cataract group experienced greater difficulty with their daily driving due to their vision.

**Table 6:** Driving Habits Questionnaire (DHQ), comparison between drivers with and without cataract.

Item	Control (% of group)	Cataract (% of group)	*p*-value
**1. Driving in the rain**			
No difficulty	50	28.6	
Difficulty	50	57.1	
I no longer drive in that situation	0	14.3	
Mean score	4.44 ± 0.62	3.86 ± 1.35	0.357
**2. Driving alone**			
No difficulty	100	71.3	
Difficulty	0	14.3	
I no longer drive in that situation	0	14.3	
Mean score	5.00 ± 0.00	4.29 ± 1.50	0.297
**3. Parallel parking**			
No difficulty	88.9	85.7	
Difficulty	11.1	14.3	
I no longer drive in that situation	0	0	
Mean score	4.89 ± 0.32	4.71 ± 0.76	0.883
**4. Left turns in traffic**			
No difficulty	94.4	100	
Difficulty	5.6	0	
I no longer drive in that situation	0	0	
Mean score	4.94 ± 0.24	5.00 ± 0.00	0.836
**5. Driving on freeways**			
No difficulty	100	100	
Difficulty	0	0	
I no longer drive in that situation	0	0	
Mean score	5.00 ± 0.00	5.00 ± 0.00	1.000
**6. Driving in heavy traffic**			
No difficulty	77.8	71.4	
Difficulty	22.2	14.3	
I no longer drive in that situation	0	14.3	
Mean score	4.78 ± 0.43	4.29 ± 1.50	0.745
**7. Driving in rush hour**			
No difficulty	77.8	71.4	
Difficulty	22.3	14.3	
I no longer drive in that situation	0	14.3	
Mean score	4.72 ± 0.58	4.29 ± 1.50	0.745
**8. Driving at night**			
No difficulty	55.6	0	
Difficulty	44.4	75	
I no longer drive in that situation	0	25	
Mean score	4.56 ± 0.51	4.58 ± 1.08	< 0.001
Composite score	94.79 ± 5.36	82.59 ± 19.42	0.025

Percentages of responses (subjects referring any level of difficulty, i.e., little, moderate, or extreme difficulty, are pooled) and mean scores are included for each item.

Point scale for mean scores: 5 = no difficulty; 4 = a little difficulty; 3 = moderate difficulty; 2 = extreme difficulty; 1 = so difficult that I no longer drive in that situation.

## Discussion

The results of visual assessments demonstrated that older drivers with cataract had significantly worse visual acuity, contrast sensitivity, and they also showed a significantly higher level of intraocular straylight, which agrees with previous studies^9,24,34^. This impairment is manifested in all visual parameters for both the worse and better eyes in our sample, given that most drivers with cataract had lens opacity in both eyes.

Drivers typically self-regulate their behavior to compensate for such limitations. Thus, the cataract group adopted lower speeds than the control group in all scenarios except scenario 3, a straight segment where they drove at similar speeds (mean difference of approximately 1.0 kph). This agrees with previous research in real^10,35,36^ and simulated driving situations^9^. Moreover, the difference in speed selection between the two groups was more accentuated in roads with a higher speed limit, such as scenarios 1 and 2, with a limit of 120 kph, and scenarios 4, 7, and 8, with a limit of 90 kph. Although this tendency was not observed in scenario 3, these results indicate that drivers with cataract may feel less safe on high speed roads. This is congruent with previous studies based on self-reported data collected from questionnaires and naturalistic driving data, which have shown that drivers affected by cataract often avoid driving on highways/freeways^2,37^ and report driving more slowly than the general traffic flow^11^. Driver self-regulation is present not only in drivers with cataract, but also in drivers with visual impairment due to other ocular disease. Glaucoma is one of them, and visual field loss result in driving self-restriction^38^. Some of the self-regulation strategies of these drivers include avoiding more demanding situations such as driving at night, in rush hour or driving in high-speed roads^39–41^. Another ocular disease that leads to driving self-regulation is age related macular degeneration (ARMD). They usually start by reducing night-time driving^42,43^ and continue avoiding other situations such as driving in rush hour, in high-speed roads or in adverse meteorological conditions^18,43,44^. The study of Szlyk et al. compared drivers with ARMD with a control group in a driving simulator and an on-road circuit. The authors found that ARMD drivers avoided unfamiliar areas or changing lanes and they adopted slower speeds than the control group^43^. In agreement with our results for drivers with cataract, these drivers seem to extreme caution with strategies such as reducing their speeds, memorizing the route, increasing the road scanning or using a passenger who verbalize details of the road^45^.

The details of the road mark its complexity and can determine the driver's behavior. In our study, road features selected for the different scenarios proved to be significant predictors of speed management. To the best of the authors’ knowledge, this is the first study into how road traffic complexity influences the speed behavior of drivers with cataract. In general, drivers modified their speed behavior under the presence of certain road features that add complexity^46^. The main cause of speed reductions was a curved layout, such as those in scenarios 1 to 6. This finding agrees with previous research in older drivers with no visual impairment^47^. On the other hand, when driving in an urban environment, participants adopted speeds well under the limit (50 kph). Contrastingly, we did not observe the same result on the mountain road at a comparable speed limit (40 kph). This may reflect that urban environments are perceived as more complex due to the presence of greater visual clutter^46,48,49^. For the inner-city section drivers need to interact with a more visual information (road signs, traffic lights, roundabouts, roadside advertising, and buildings) and more road users (pedestrians, moving or parked cars). Visual impairment when driving along city road could therefore generate a greater feeling of insecurity in drivers^48^.

Apart from environment complexity, some driver characteristics were also significant predictors of driving speed. Our sample consisted exclusively of older drivers, so we could not assess whether age had a significant effect. However, other works have found that older drivers adopt lower speeds than younger ones, in an attempt to compensate for declines in their motor, cognitive, and sensory capacities^35,36,46–48^. Gender was a significant predictor of speed management, with men adopting higher speeds. Gwyther and Holland (2012) demonstrated that a high proportion of women of all ages avoid high-speed roads, while this behavior is much less frequent among men^50^. Research has also revealed that men are greater risk takers, as they are more likely to adopt behaviors such as speeding^51–53^. Among visually impaired older drivers, women are known to self-regulate their driving to a greater extent^37^. Nevertheless, it should be taken into consideration that our sample contained fewer women than men, so further research is necessary to confirm this finding.

Of the visual tests used, intraocular straylight in the better eye was a significant predictor of speed behavior. This is the first time that this parameter has been associated with self-regulation patterns in older drivers. Other authors have reported the importance of scattering-related visual parameters (i.e., straylight or disability glare) in drivers, proposing a straylight cut-off value of 1.4 for safe driving^24,32,33^. Our recent studies have shown that higher levels of intraocular scattering are associated with a poorer simulated driving performance in drivers of different ages and older drivers with and without cataract—in fact, it is a better predictor of driving ability than the visual acuity test^9,29,54^. The intraocular straylight parameter could provide an accurate quantification of the detrimental effects of intraocular scattering on visual perception, being particularly important for people with cataract^55^. Nevertheless, at the time of writing we lack a standardized method to assess the effects of intraocular scattering. Although several glare assessment devices have been used in previous studies, these devices base their evaluations on the effect that glare has on visual perception (i.e., they measure the effect of glare on visual acuity or contrast sensitivity), while they do not appear to correctly assess the conditions that can affect driving performance^55^. In fact, for older drivers, different authors have not found any relationship between the results obtained with this type of glare tests and a poorer driving performance^10,36,56,57^, a higher risk of accidents^8,58,59^, or a greater level of self-reported driving difficulty and self-regulation patterns^60,61^.

On the other hand, contrast sensitivity has proved to be a non-significant predictor of speed management. However, the driving environment comprises stimuli of different contrast levels, some of them are low contrast, so drivers need good contrast sensitivity in order to drive safely^33^. Owsley et al. found that impaired contrast sensitivity was the only predictor of crash involvement in drivers with cataract, with a stronger correlation with the worse eye^8^. Furthermore, contrary to our result, Agramunt et al. and Fraser et al. demonstrated that reduced contrast sensitivity is associated with driver self-regulation in drivers with bilateral cataract^19,37^. Similarly, on-road studies including drivers with cataract have also highlighted the importance of CS as a predictor of driving performance^10,36^. However, some of these differences could be due to the reduced number of subjects with cataract who were included in our study. After cataract surgery, the increase in contrast sensitivity is associated with improved driving performance^10^ and a reduced likelihood of self-regulation^19,62^.

The role of contrast in the driving environment and driver’s contrast sensitivity for speed judgment and selection has been widely discussed. Cataract increases straylight, and this causes a veil of luminance over the retina that importantly reduces the retinal image contrast. According to our findings, this results in lower selected speeds. Studies involving video-based driving simulator scenes with reduced contrast have indicated that, under such conditions, drivers perceive speeds as slower than actual speeds and, as a result, may adopt faster speeds^63,64^. However, the study by Owens et al. on a closed-road circuit found opposite results, with drivers estimating their speeds as faster and selecting lower speeds under reduced contrast conditions^65^. In the same line, there is simulator-based research, such as the present study, which demonstrates reductions in speed under conditions of poorer contrast sensitivity. For example, speed self-regulation in drivers under the influence of cannabis have shown to be significantly predicted by contrast sensitivity. Drug-impaired drivers demonstrated lower contrast sensitivity and selected lower speeds^31^. In an attempt to clarify the cause of the discrepancy between studies, Brooks and Rafat included three-dimensional information in the video-based speed judgment task. In these circumstances, speed was also estimated to be slower under reduced contrast^66^. In view of this result, it has been suggested that the cause of the underestimation of speed could be the reduced visual field provided by the driving simulator. The aforementioned studies on driving simulator used video clips to show the driving scene with a restricted field of view (25–38°)^63,66^ whereas our driving simulator provides 180°, thus better simulating the real driving context. It is also worth noting that deficiencies in speed perception could be counteracted by better control of the speedometer. When visibility is reduced by fog, drivers have shown to check their speedometer less often because they avoid taking their attention away from the road^64^. The same could be true for drivers with cataract, who may have an additional difficulty, a poor view of the speedometer, forcing them to take their eyes off the road for longer periods of time to check it. In our study, the speedometer was easily accessible on the central display, with an appropriate font size. Nevertheless, Owens et al. evaluated drivers without seeing the speedometer, and they perceived that they were travelling faster^65^. Future studies should measure the proportion of time drivers spend looking at the speedometer as a possible influencing factor.

Other studies have also found results along the same lines as the findings of the present work, highlighting the importance of non-standardized visual tests. For instance, visual attention and oculomotor efficiency are latent variables of interest for the driving task, as indicated by the study of Gené-Sampedro et al. The authors found that drivers performed better than non-drivers in the Adult Development Eye Movement test (ADEM). In addition, age was a significant predictor of the test performance, which worsened with increasing age^67^. This aspect could also be impaired in drivers with cataract, making it more difficult to integrate information during driving, thus potentially triggering driver self-regulation. Furthermore, in line with our results, Bal et al. reported that straylight and, to a lesser extent, contrast sensitivity are complementary tests to visual acuity, as their consideration is important in terms of programing surgery and driving legality^24^. On the other hand, West et al. studied 629 drivers aged over 55 years and found an association between vision-related self-restricted driving and some visual tests such as acuity at different contrast levels or in the presence of glare^4^. Their results show that the subjective perception of visual disability could also be better predicted by tests other than the commonly used visual acuity test.

In a similar vein, our results of the DHQ show that drivers with cataract perceive greater visual difficulties when driving. It is important to note that all the participants who reported that they no longer drove in certain situations had cataract. As such, 27% of the total sample indicated they self-regulated. Other authors have obtained greater percentages of drivers with cataract who self-regulate (~ 40%)^2,37^, possibly because a visual acuity below the legal limit for driving was not an exclusion criteria in their studies. In our study, driving at night was the most commonly avoided situation, in agreement with previous works^2,34,37^. Other commonly avoided situations are driving in heavy traffic or at rush hour^2,34,37^. Some studies also indicate that these drivers with cataract tend to avoid high-speed roads such as freeways^2,37^. None of the participants in our study indicated that they avoided driving on freeways, although results from the driving simulator showed that they drove much slower than the control group in sections with a high speed limit.

This study shows some promising results with regard to the visual assessment of drivers with cataract; however, the results should be interpreted cautiously given several methodological limitations. Firstly, the sample size was limited due to the difficulty enrolling older drivers who are both active drivers and free from any eye diseases other than cataract. Moreover, simulator sickness is a very common symptom in older participants, which also hinders the recruitment of larger sample sizes^68^. Lastly, the use of a driving simulator cannot replicate the true nature of actual driving, although it can be used to assess the impact of cataract in a safe manner. However, driving simulators offer a reproducible method and the opportunity to choose specific features in the environment, which was a very important factor in our study^12^.

## Conclusions

Visual impairment due to cataract leads to a modification of some driving patterns. This study has objectively demonstrated this point by observing the effect on speed behavior. Road complexity influences driving speed, and drivers with cataract manage the road environment differently from those with no visual impairment. Our results show that speed selection correlates with gender and straylight level in the better eye. The DHQ also showed that drivers with cataract perceived greater difficulty driving due to their visual impairment, and a significant proportion avoid some challenging situations. This work could help us appreciate the mechanisms through which age-related visual impairment results in safety–critical events, and how interventions and awareness campaigns could be developed to help drivers with this condition adopt the actions needed to ensure safe driving while awaiting cataract surgery. Moreover, the results of this study could aid road safety policy makers and licensing authorities, who mainly rely on visual acuity tests even though this visual parameter may have limited value in older drivers, particularly when they have certain eye diseases such as cataract that limit their visual capacity.

## Data Availability

The datasets generated during the current study are available from the corresponding author on reasonable request.
